# Targeting *MYCN* in Pediatric and Adult Cancers

**DOI:** 10.3389/fonc.2020.623679

**Published:** 2021-02-08

**Authors:** Zhihui Liu, Samuel S. Chen, Saki Clarke, Veronica Veschi, Carol J. Thiele

**Affiliations:** ^1^ Pediatric Oncology Branch, Center for Cancer Research, National Cancer Institute, Bethesda, MD, United States; ^2^ Department of Surgical, Oncological and Stomatological Sciences, University of Palermo, Palermo, Italy

**Keywords:** MYCN, Super-enhancer (SE), cofactor, cancer, pediatric cancer, MYC, transcription factor

## Abstract

The deregulation of the *MYC* family of oncogenes, including *c-MYC*, *MYCN* and *MYCL* occurs in many types of cancers, and is frequently associated with a poor prognosis. The majority of functional studies have focused on *c-MYC* due to its broad expression profile in human cancers. The existence of highly conserved functional domains between *MYCN* and *c-MYC* suggests that *MYCN* participates in similar activities. *MYC* encodes a basic helix-loop-helix-leucine zipper (bHLH-LZ) transcription factor (TF) whose central oncogenic role in many human cancers makes it a highly desirable therapeutic target. Historically, as a TF, MYC has been regarded as “undruggable”. Thus, recent efforts focus on investigating methods to indirectly target MYC to achieve anti-tumor effects. This review will primarily summarize the recent progress in understanding the function of *MYCN*. It will explore efforts at targeting *MYCN*, including strategies aimed at suppression of *MYCN* transcription, destabilization of MYCN protein, inhibition of *MYCN* transcriptional activity, repression of MYCN targets and utilization of *MYCN* overexpression dependent synthetic lethality.

## Introduction

*MYCN* is a member of the *MYC* family of oncogenes, which also includes *c-MYC* and *MYCL* (1). *MYCN* was first reported in 1983 as an amplified gene homologous to v-*myc* in human neuroblastoma (2, 3). Like *c-MYC*, the *MYCN* gene encodes a basic helix-loop-helix-leucine zipper (bHLH-LZ) protein named N-Myc or MYCN. MYCN and c-MYC exhibit high-structural homology, including highly conserved Myc boxes (MB) and a BR-HLH-LZ motif (1, 4, 5). Both MYCN and c-MYC heterodimerize with MAX to bind to an enhancer-box (E-box) sequence with a consensus CAC(C/A)TG motif to regulate gene transcription (1, 4–6). *MYCN* and *c-MYC* differ in their expression patterns and regulation. While *c-MYC* is ubiquitously and highly expressed in most rapidly proliferating cells throughout development and in adult tissues, *MYCN* is preferentially expressed in neural tissues including the forebrain and hindbrain, as well as pre-B cells, cells in the intestine, heart and kidney during embryogenesis (5, 7). Tissue-specific conditional deletions demonstrated that *c-MYC* is necessary for the development and growth of specific hematopoietic cell lineages, crypt progenitor cells in the intestine and many other types of cells where *c-MYC* is expressed (8). *MYCN* but not *c-MYC* is essential during neurogenesis for the rapid expansion of progenitor cells and the inhibition of neuronal differentiation (9). Importantly, investigations at a gross level indicate that *Mycn* can substitute for *c-Myc* in murine development (10). For example, transgenic expression of *Mycn* from the *c-Myc* locus (*c-Myc^N/N^*) rescues the embryonic lethality associated with the loss of *c-Myc.* Unlike *c-Myc* gene that is expressed throughout lymphocyte development, *Mycn* is only expressed in the precursor stage lymphocyte of development (10, 11), but *Mycn* can replace all *c-Myc* functions required for lymphocyte development in the *c-Myc^N/N^* mice (10). However, subtle differences between *c-Myc^N/N^* and normal mice were observed, such as the observation of periodic skeletal muscle dystrophy in some newborn *c-Myc^N/N^* mice (10). This indicates a general functional similarity between these TFs in regulating certain lineages of murine cell growth and differentiation during embryogenesis and late development.

As TFs, both MYCN and c-MYC directly regulate transcription of genes that are involved in the control of cell growth, the cell cycle, proliferation, survival, apoptosis, pluripotency, self-renewal, DNA replication, RNA biology, metabolism, metastasis, angiogenesis and immune surveillance to play an oncogenic role (5, 12). Most studies indicate that MYC regulates differential gene transcription in the majority of cell types and model systems (4, 12–14). However, instead of regulating differential gene transcription, it has been shown that in B cells, the high levels of c-MYC expression results in a global increase in mRNA levels during the mitogenic stimulation of early B cells (15). Similarly, the expression of high levels of c-MYC in tumor cells leads to an increase in total levels of transcripts in each cell (16). These studies conclude that high levels of c-MYC amplify transcriptional output (15, 16). Further studies and analyses of existing data reveal that MYC dependent changes in global RNA levels may occur only when the cells are cultured under special conditions and/or after prolonged MYC activation. It is possible that a feedback effect from MYC-induced physiological and metabolic changes contributes to a global RNA amplification (4, 12, 13). MYCN interacts with Transcription Factor IIIC complex (TFIIIC), DNA topoisomerase II alpha (TOP2A) and the cohesion complex component RAD21, but in S phase, Aurora-A kinase displaces these interactors from MYCN to block MYCN-dependent promoter release of RNA polymerase II to suppress MYCN-dependent gene transcription (17). MYCN recruits BRCA1 to promoter-proximal regions, stabilizing mRNA de-capping complexes. This enables MYCN to suppress R-loop formation in promoter-proximal regions and prevent MYCN-dependent accumulation of stalled RNAPII, thus, enhancing MYCN transcriptional activation (18). The discovery of this non-canonical transcriptional function of MYCN may explain the discrepancy between universal binding and the small effects on relative and/or absolute mRNA levels of most genes that are bound by the MYC proteins (4, 18).

This review discusses *MYCN* genetic alterations in different types of cancers, the structure and transcriptional function of MYCN and the strategies used to target *MYCN* indirectly.

### *MYCN* Is an Oncogenic Driver in Many Types of Cancers

Deregulation of *MYCN* occurs in both pediatric cancers and adult cancers. *MYCN* amplification has been found in pediatric cancers including neuroblastoma, rhabdomyosarcoma, medulloblastoma, Wilms tumor and retinoblastoma. Amplification of the *MYCN* oncogene is present in 18–20% of all neuroblastomas (40% of high-risk neuroblastomas) and is an adverse prognostic factor (19–23). In alveolar rhabdomyosarcoma, amplification of *MYCN* is present in 25% of cases and overexpression of *MYCN* occurs in 55% of cases (24, 25). Amplification of *MYCN* is observed in 5–10% of medulloblastomas and is associated with poor prognosis (26–28). Copy number gains that include the *MYCN* locus are detected in 12.7% of Wilms tumors and 30.4% of diffuse anaplastic Wilms tumors, and *MYCN* gain is associated with poorer relapse-free and overall survival (29). In retinoblastomas, *MYCN* amplification is present in <5% of patients, and *MYCN* gain is associated with poor prognosis (30, 31). In adult cancers, amplification of *MYCN* is present in 40% of neuroendocrine prostate cancers and 5% of prostate adenocarcinomas (32), 15%-20% of small-cell lung cancers (33, 34) and 17.5% of basal cell carcinomas (35). Overexpression of *MYCN* is present in a subset of T-cell acute lymphoblastic leukemias (36), glioblastoma multiforme (37, 38) and breast cancer (39). Importantly, the amplification or overexpression of *MYCN* in the majority of these adult cancers is found to be associated with a poor prognosis.

To investigate whether *MYCN* functions as an oncogenic driver, genetically engineered mouse models (GEMM) have been generated to express *MYCN* in specific cell lineages. The transgenic expression of *MYCN* in the neural crest lineage of mice or zebrafish alone, or in combination with *LMO1* or activated *ALK* gives rise to neuroblastomas (40–44). The transgenic expression of *MYCN* in murine luminal prostate epithelial cells in combination with *Pten* knockout results in a GEMM model with neuroendocrine prostate cancer formation (45). Mice transplanted with bone marrow expressing *MYCN* developed clonal and transplantable acute myeloid leukemias (46). When neural stem cells (NSCs) from different brain regions are transduced with a protein stabilizing MYCN(T58A) mutation and transplanted into their homotypic regions they give rise to distinct tumors. The transplantation of forbrain MYCN(T58A) NSCs gives rise to gliomas (47), while cerebellum and brain stem MYCN(T58A) NSCs transplants give rise to medulloblastoma and primitive neuroectodermal tumors (47). The enforced expression of *MYCN* in primary cerebellar granule neuron precursors isolated from *Ink4c*(-/-), *p53*(-/-) mice also results in medulloblastomas when transplanted into the brains of immunocompromised mice (48). These studies demonstrate that *MYCN* functions as an oncogene and is capable of driving tumor formation in cells with different lineage specific genetic programs to give rise to distinct tumor types. Thus, the inhibition of *MYCN* will be an important anti-tumor therapeutic strategy in many different human cancers with aberrantly over-expressed *MYCN*.

### MYCN Structure: Critical Regions That Mediate Protein-Protein Interaction and Transcriptional Activity

**Figure 1:**
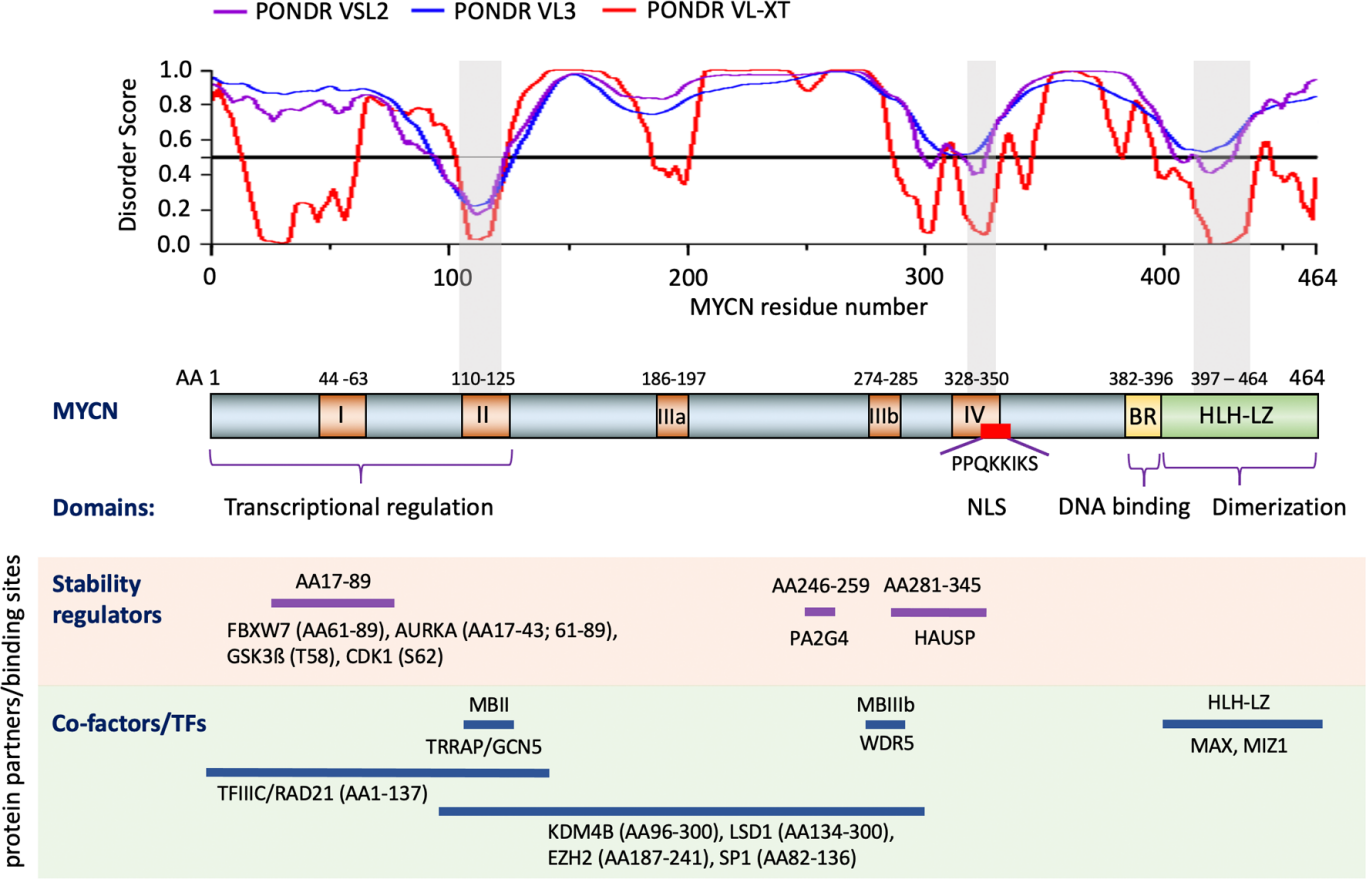
Structure and functional domains of MYCN. Three predictors of the intrinsically disordered region prediction tool PONDR are used to identify intrinsically disordered regions of MYCN (top section). Functional domains of MYCN defined by comparing c-MYC and mutagenesis assay (middle section). Examples of known MYCN protein partners and the regions of MYCN that contributed to the interaction (bottom section). Notes: Color boxes on the MYCN protein diagram: brown box, Myc homology Box (MB) I-IV; yellow box, Basic Region (BR); green box, Helix-Loop-Helix-Leucine Zipper (HLH-LZ); red box, nuclear localization signal (NLS). Gray shade box on the disorder score graph and MYCN protein diagram, regions of MYCN with relatively low disorder score. AA: amino acid.

MYCN is composed of 464 amino acids (AA) with several functional domains (**Figure 1**) derived from sequence homology to known c-MYC protein functional domains (NCBI reference number of MYCN, NP_001280157.1 verse c-MYC, CAA25015.2) and mutagenesis analyses (1, 6). The N-terminal transcriptional regulation domain contains two highly conserved regions known as Myc Homology Box (MB), MBI and MBII. The central region contains 3 more MBs with a nuclear localization signal that overlaps MBIV. The C-terminal basic region (BR) is involved in DNA binding while the HLH-LZ heterodimerizes with Max (1). Many critical proteins regulating various biological processes do not have unique structures, but contain intrinsically disordered regions (IDRs), making this structural region extremely dynamic (49, 50). IDRs are involved in modulation of the specificity or affinity of protein binding interactions (49, 50). The IDRs in a protein can undergo characteristic disorder-to-order transitions upon interactions with specific binding partners and/or through post-translational modifications (49). Using a protein intrinsic disorder region prediction tool PONDR (http://www.pondr.com/) (51) to analyze MYCN, we find that the majority of MYCN residues tend to form broad disordered regions (**Figure 1**), which indicates that MYCN has the potential to bind to many different partners. Moreover, the intrinsically disordered character of MYCN suggests that using it as a direct drug target would be challenging due to its structural flexibility.

Critical regions within MYC/MYCN proteins have been implicated in regulating protein stability. Residues Ser62 (S62) and Thr58 (T58) within MBI are critical phosphorylation sites for MYC/MYCN protein stability during cell cycle progression. As growth factors stimulate cell progression through the cell cycle, protein stability is tightly regulated. Phosphorylation at S62 of MYCN protein is mediated *via* CDK1, which stabilizes MYCN and primes T58 for phosphorylation by GSK3β. GSK3β is repressed by phosphatidylinositol 3-kinase (PI3K) and AKT kinase signaling (52–57). Dephosphorylation of MYC-S62 *via* protein phosphatase 2A (PP2A) enables E3 ligase FBXW7 binding to phosphorylated MYC-T58, targeting it for ubiquitination and subsequent degradation by the proteasome (58, 59). The regulation of MYCN protein stability is cell-cycle dependent. In normal neuronal progenitors, CDK1 phosphorylates MYCN protein at S62 in G1-phase. As cells enter M-phase, signaling by growth factors declines leading to activation of GSK3β enabling phosphorylation of MYCN(T58) which leads to its degradation (52). Two additional ubiquitin ligases, TRIM32 and HUWE1, are involved in regulation of MYCN degradation. During late M-phase, the ubiquitin ligase TRIM32 is bound to the mitotic spindle pole apparatus in conjunction with MYCN, contributing to its ubiquitination and degradation (60). HUWE1, a HECT-domain E3 ubiquitin ligase, binds to MYCN and primes it for MYCN-K48-linked polyubiquitination and proteasomal-mediated degradation (61, 62).

MYCN protein degradation is antagonized through interactions with different proteins at distinct MYCN regions (**Figure 1**). Aurora A kinase (AURKA) associates with the mitotic spindle poles and interacts with the N-terminus of MYCN in cells over-expressing MYCN and in this way interferes with FBXW7-mediated degradation leading to MYCN stabilization (17, 56, 63). The ubiquitin-specific protease HAUSP binds to a partially overlapping region of MBIII and MBIV in MYCN, but not c-MYC (**Figure 1**), to specifically deubiquitinate MYCN, which results in MYCN protein stabilization (64). The proliferation-associated 2AG4 protein (PA2G4) directly binds to and stabilizes MYCN by protecting it from ubiquitin-mediated proteasomal degradation (65). Co-immunoprecipitation results show that a MYCN deletion mutant (AA82–254) binds strongly to PA2G4, and further studies show that a 14 AA MYCN oligopeptide (AA246–259) sequence contributes to this protein-protein interaction (65). Additionally, MYCN has been found to be methylated at R160, R238 and R242; protein arginine methyltransferase 5 (PRMT5) physically interacts with MYCN and increases MYCN protein stability, possibly by methylating MYCN at R242 (66). The identification of different signaling pathways and proteins regulating MYCN protein stability provides additional modes for indirect targeting of MYCN.

TFs, co-repressors and co-activators interact with different regions of MYCN (**Figure 1**), enabling MYCN to activate or repress gene transcription. MYC family proteins directly interact with MAX through HLH-LZ to form a heterodimer and activate transcription by binding to E-box elements (1, 6). Activation involves the recruitment of multiple coactivators and protein complexes to E-box elements. The TIP60 acetyltransferase complex and the histone acetyltransferase GCN5 are bound to MYC indirectly through the TRRAP adaptor protein that interacts with MBII of the MYC protein (67–70). Two other proteins, TIP48 and TIP49, found in the TIP60 complex, are involved in chromatin remodeling and bind to the N-terminus of MYCN (67). Recent studies show that target gene recognition by c-MYC and MYCN depends on its interaction with the histone H3-K4-methyl-associated protein WDR5 and their interaction is mediated through the MBIIIb region of c-MYC (71, 72). MYCN interacts with the H3K9me3/me2 demethylase KDM4B through the region between AA96-300 and when overexpressed MYCN recruits KDM4B to E-box regions to decrease H3K9me3 levels (73). Thus, MYCN may also activate gene transcription by relieving transcriptional repression. Moreover, MYCN AA1-137 also interacts with TFIIIC and RAD21 to regulate the pause release of RNA Polymerase II (17). BRCA1 interacts with MYCN and enables MYCN to suppress R-loop formation in promoter-proximal regions, thus enhancing transcription (18). When MYCN functions as a transcriptional repressor, it interacts with SP1 and MIZ1 to repress gene transcription (74–77). The region between MYCN AA82-136 that includes the MBII domain specifically interacts with SP1 in pull down experiments, whereas MYCN AA400-464 that includes the HLH-LZ domain interacts with MIZ1 (74). These MYCN/SP1/MIZ1 interactions repress gene transcription by recruiting HDAC1 (74). MYCN, *via* MBIII, associates with EZH2, a methyltransferase and member of the polycomb repressor complex 2, to suppress gene transcription (78). Similarly, MYCN physically binds lysine-specific histone demethylase 1A (KDM1A/LSD1) through MBIII to repress gene transcription (79). The above studies show how MYCN interacts with the epigenome to regulate gene transcription.

### Targeting *MYCN* Transcription

**Figure 2:**
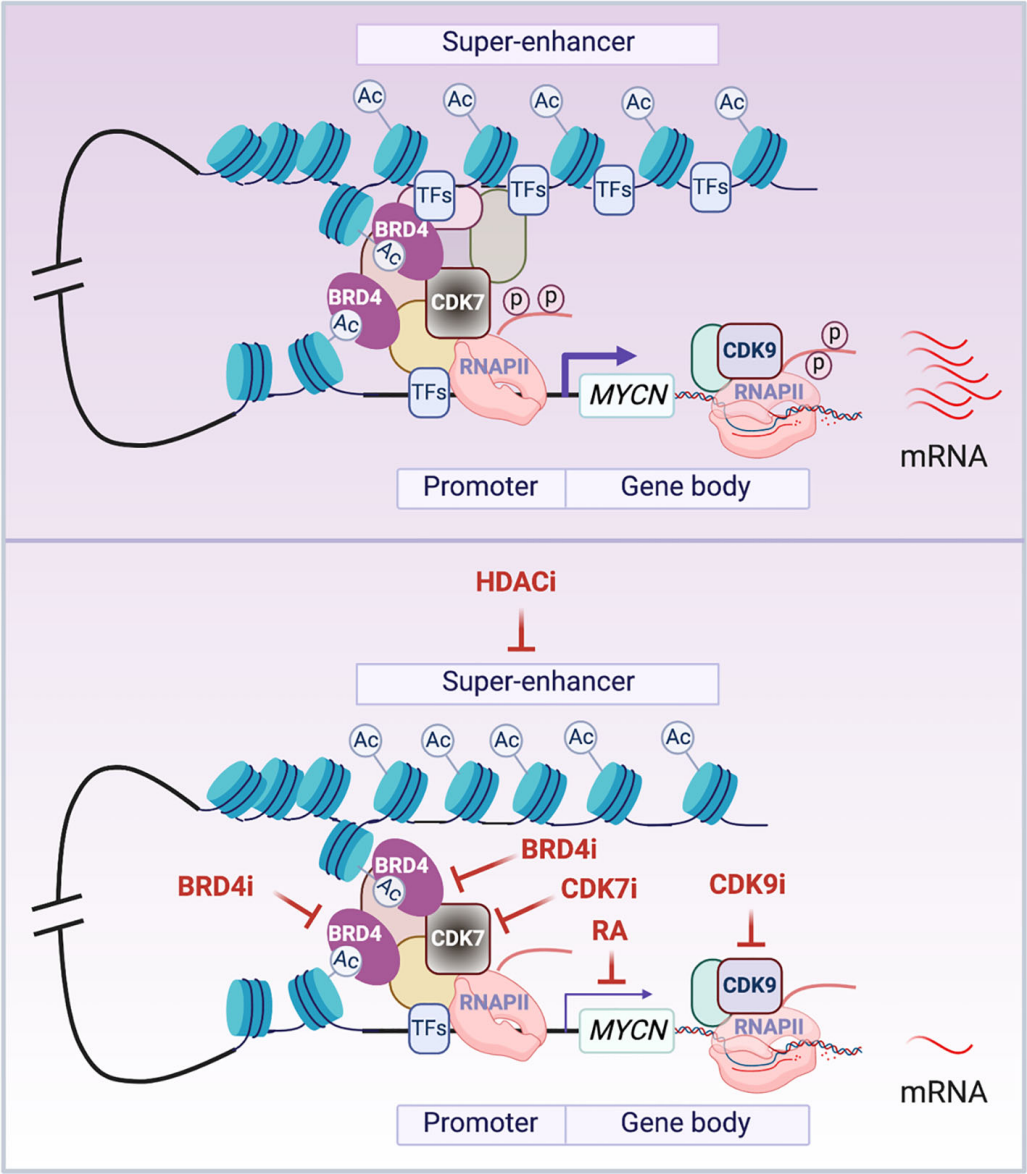
Transcriptional regulation of *MYCN*. The schematic illustrates the presumed looping between the super-enhancer (SE) and the promoter of *MYCN* gene. In cancer cells, MYCN is driven by SEs that are marked by stretches of acetylated lysine 27 of histone 3 (H3K27Ac). BRD4 is a chromatin ‘reader’ that binds to acetylated lysine residues (AcK) and activates *MYCN* transcription. CDK7 is a TFIIH subunit that phosphorylates the carboxy-terminal domain of RNA Pol II (RNAPII) to initiate *MYCN* gene transcription. CDK9 is a pTEFb subunit that phosphorylates the carboxy-terminal domain of RNAPII to regulate *MYCN* transcriptional elongation. The enrichment or activation of these components of the transcriptional machinery in cancer cells results in aberrantly elevated transcription of *MYCN* (top panel). The treatment of cells with HDAC inhibitors (HDACi) inactivates *MYCN* SEs possibly through disrupting normal looping and depleting transcription factors (TFs) that bind to the SEs; BRD4 inhibitors (BRD4i) impact the ‘reader’ function of BRD4 to inactivate *MYCN* gene transcription; CDK7 inhibitors (CDK7i) and CDK9 inhibitors (CDK9i) treatment impedes the phosphorylation of RNAPII to inhibit *MYCN* gene transcription initiation and elongation; RA treatment inactivates *MYCN* transcription in a RA response element independent manner (bottom panel). Notes: circled ‘Ac’ represents H3K27Ac; circled ‘p’ represents phosphate at the RNAPII tail.

Many mechanisms have been identified to be involved in the transcriptional regulation of *MYCN* (**Figure 2**). Soon after the discovery of the *MYCN* gene, it was found that retinoic acid (RA) treatment of NB cells resulted in a down-regulation of *MYCN* expression at the mRNA level, and this preceded cell cycle arrest and implementation of a differentiation program (80). This indicated that *MYCN* down-regulation, at least partially, contributes to the biological effect of RA on NB cells. A classic RA response element (RARE) was not implicated in RA regulation of *MYCN* transcription, as studies showed that RA exerts its effects across multiple regulatory regions within the *MYCN* promoter, distally or even on different chromosomes (81). Retinoid repression of MYCN transcription was a major motivation for the inclusion of 13 cis-retinoic acid during the consolidation phase of treatment for high-risk neuroblastomas (82). Retinoid regulation of *MYCN* represents one of the first strategies developed to target *MYCN* gene transcription and provides an example of indirect targeting of *MYCN*.

Gene transcription is mediated by cis-regulatory elements such as enhancers and promoters. Enhancers are distal regulatory elements in the genome that play an important role in driving cell-type-specific gene expression and are frequently mis-regulated in cancer (83, 84). Super-enhancers (SEs) are composed of a cluster of enhancers that are central to the maintenance of cell identity in normal development and disease (85). SEs were found to be associated with various oncogenic molecules including both *c-MYC* and *MYCN*; this makes them putative therapeutic targets for cancer therapy (86–89). Histone deacetylases (HDACs) have an important function in regulating both DNA packaging in chromatin and gene transcription. Treatment of NB cells with HDAC inhibitors such as MS-275, BL1521 or SAHA resulted in a decrease in *MYCN* mRNA levels accompanied by cell apoptosis (90–92). Although not directly demonstrated, recent studies have shown that HDAC inhibition results in enhancer remodeling and suppression of oncogenic SEs possibly through disruption of normal chromatin-looping and TFs depletion on the SEs (93, 94). This may be involved in the HDAC inhibitor mediated repression of *MYCN* transcription (**Figure 2**).

*MYC*-driven tumors are especially sensitive to inhibition of BET bromodomain containing proteins (BRD1–4) (95). BRD4 belongs to family of proteins that contain variable numbers of bromodomains and a central ET domain and function as chromatin “readers” by binding to acetylated lysine residues (**Figure 2**). BRD4 has also been implicated in regulating RNA-PolII transcriptional activity (96). BET inhibitors downregulate *c-MYC* transcription, suppress *MYC*-dependent target genes and inhibit myeloma cell proliferation (95). An unbiased screen of 673 genetically characterized tumor-derived cell lines shows that neuroblastoma cell lines with *MYCN* amplification are more sensitive to JQ1 treatment compared to *MYCN*-wild-type tumors. BRD4 knock-down phenocopied these effects, indicating that BRD4 functions as a transcriptional regulator of *MYCN*. Importantly, BET bromodomain-mediated inhibition of *MYCN* suppresses neuroblastoma growth both *in vitro* and *in vivo* (97). Similarly, OTX015 (Oncoethix), a small molecule that prevents BRD2/3/4 from binding to acetylated histones, also represses *MYCN* transcription. This study showed that BRD4 binds to super-enhancers (SEs) and *MYCN* target genes, while OTX015 treatment disrupts BRD4 binding and transcription of *MYCN* as well as its target genes (98), which is consistent with the finding that bromodomain inhibitor treatment selectively inhibits oncogenes by disrupting their SEs (99). Importantly, bromodomain and extra-terminal domain inhibitor (BETi), GSK525762, is under phase I clinical trial for solid tumors including NB (100). In addition to *MYC*, many tumor-associated genes such as *RUNX1, FOSL2, BCL3* and *ID2* are driven by SEs in diverse tumor types (99). However, SEs drive many cell identity genes essential for normal cell development, such as Oct4, Sox2 and Nanog in embryonic stem cells (101), so as with many cytotoxic agents a therapeutic window is needed when using BETi for the treatment of cancer patients to minimize side effects. Recent studies showed that the combination of a bromodomain inhibitor with a CDK7 inhibitor, an AURKA inhibitor or an HDAC inhibitor is significantly more effective in suppressing *MYCN*-driven NB tumor growth than either drug alone (88, 102, 103). This highlights the importance of combinatorial therapeutic approaches for cancer treatment.

To regulate gene transcription, the RNA polymerase II (RNAPII) transcription initiation apparatus needs to be recruited to promoters by specific DNA binding transcription factors. Promoter-proximal pausing of RNAPII is a post-initiation regulatory event, and c-Myc plays a key role in release of Pol II at many actively transcribed genes in ES cells (104). Cyclin-dependent kinases (CDKs) are important in regulating the transcription cycle of RNAPII. The TFIIH subunit CDK7 and the pTEFb subunit CDK9 phosphorylate the carboxy-terminal domain of RNAPII, facilitating efficient transcriptional initiation, pause release and elongation. This suggests that the inhibition of these CDKs would be expected to block *MYC*-driven transcriptional amplification. Indeed, THZ1, a covalent inhibitor of CDK7, was found to selectively target *MYCN*-amplified NB cells, leading to global repression of *MYCN*-dependent transcriptional amplification and reductions in expression of SE-associated oncogenic drivers including *MYCN* itself and suppression of NB tumor xenograft growth (87). CYC065, an inhibitor of CDK9 and CDK2, was found to selectively target MYCN-amplified NB cells by leading to a selective loss of nascent MYCN transcription (105). These studies indicate that the inhibition of CDK7 or CDK9 can be exploited to disrupt aberrant *MYCN*-driven transcription and to repress *MYCN* gene transcription as a therapeutic for *MYCN*-driven cancers (**Figure 2**).

DNA G-quadruplexes (G4s) are noncanonical DNA structures that are formed by guanine-rich DNA sequences. They often occur in the promoter regions of oncogenes and regulate their expression (106–108). An early study identified a specific G4 structure formed in the *c-MYC* promoter region. Although a cationic porphyrin TmPyP4, which binds non-selectively to G4s *in vitro* was able to inhibit the transcription of c-*MYC* (108), a recent study identified a small molecule DC-34 that more specifically binds to the c-MYC G4 *in vitro.* In a G4-dependent mechanism, DC-34 plays a more potent and selective role in downregulating *MYC* gene transcription compared to other G4 containing oncogenes in leukemia cells. Moreover, the treatment of cancer cells with DC-34 results in a G0-G1 arrest and a reduction of cell viability (106). Although not yet reported, the identification and targeting of G4s within the *MYCN* promoter and regulatory regions would be another approach to inhibit *MYCN* gene transcription.

Many TFs have been validated as oncogenes in human cancers and their dysregulated transcriptional programs result in a high dependency of cancer cells on these gene expression regulators (109). Importantly, RA, inhibitors of HDACs, BET bromodomain containing proteins, CDK7, CDK9 and small molecules that bind to G4s have been demonstrated to be effective for the treatment of many types of cancers by targeting their dysregulated transcriptional programs (109, 110). Thus, targeting *MYCN* at branch points involved in its oncogenic regulation of transcription (**Figure 2**) is an important therapeutic approach for *MYCN*-driven cancers.

### Targeting MYCN Protein Stability

**Figure 3:**
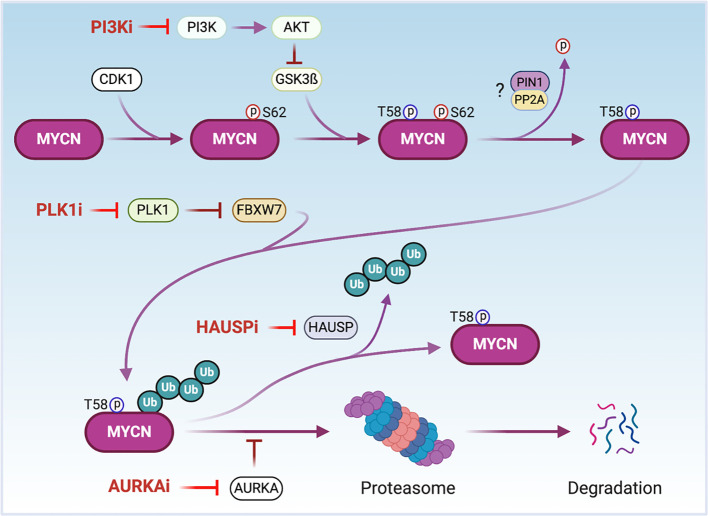
The regulation of MYCN protein stability. CDK1 phosphorylates MYCN at serine 62 (S62) to stabilize MYCN and prime threonine 58 (T58) for phosphorylation *via* GSK3β. AKT phosphorylates GSK3ß inactivating its kinase. After dephosphorylation of S62 possibly through PIN1/PP2A, MYCN is poly-ubiquitinated by the ubiquitin ligase FBXW7 and undergoes proteolytic degradation *via* the proteasome. AURKA binds to and stabilizes phosphorylated and poly-ubiquitinated MYCN to protect MYCN from degradation. PLK1 destabilizes FBXW7 to counteract FBXW7-mediated degradation of MYCN. The ubiquitin-specific protease HAUSP deubiquitinates MYCN to stabilize it. Thus, the treatment of cells with PI3K, AURKA, PLK1 or HAUSP inhibitors (PI3Ki, AURKAi, PLKi or HAUSPi) leads MYCN proteasomal degradation. Notes: circled ‘p’ represents phosphate; circled ‘Ub’ represents ubiquitin.

MYCN is a short-lived protein whose stability is tightly regulated by different signaling pathways that target it for ubiquitin-mediated degradation by the proteasome (52, 55, 111) (**Figure 3**). A major signaling pathway affecting MYCN protein stability occurs upon activation of PI3K. PI3K activates Akt which phosphorylates GSK3ß, suppressing GSK3ß kinase activity. This results in decreased phosphorylation of MYCN-T58 which is critical for targeted degradation by the proteasome (55) (**Figure 3**). As expected, inhibitors of PI3K destabilize the MYCN protein and suppress tumor growth in the TH-MYCN GEMM NB model (53, 112). In NB cells, AURKA interacts with MYCN by interfering with the FBXW7 subunit of the ubiquitin protein ligase complex to impede MYCN ubiquitination and subsequent degradation (56) (**Figure 3**). Treatment with AURKA inhibitors decreases MYCN protein levels resulting in suppression of NB tumor growth, making AURKA a suitable target for *MYCN*-driven cancers (32, 113–117). Due to the promising preclinical results, the oral AURKA inhibitor MLN8237 is under clinical evaluation for multiple cancers including relapsed NB (118). PLK1 is a serine/threonine kinase formally known as the polo-like kinase. The PLK1 inhibitor BI 2356 exhibits strong antitumor activity in NB cells *in vitro* and *in vivo* (119). PLK1 does not directly bind to the MYCN protein. Rather, it increases MYCN protein stability by destabilizing the FBXW7 ubiquitin ligase complex to counteract FBXW7-mediated degradation of MYCN (120) (**Figure 3**). Importantly, *MYCN*-amplified tumor cells in neuroblastoma and small cell lung cancer are more sensitive to treatment with PLK1 inhibitors than tumors with normal MYCN copy number, indicating that PLK1 inhibitors are potential therapeutics for *MYCN*-overexpressing cancers (120).

Components of the proteasome targeting and degrading system contribute to MYCN protein regulation. The ubiquitin-specific peptidase HAUSP (also known as USP7) binds to and deubiquitinates MYCN leading to its stabilization (64) (**Figure 3**). HAUSP is highly expressed in tumors from NB patients with poor prognoses. Silencing of HAUSP expression in NB cells destabilizes MYCN and results in an inhibition of MYCN mediated functions. Importantly, the HAUSP inhibitor P22077 markedly suppresses the growth of MYCN-amplified human neuroblastoma cell lines in xenograft mouse models (64).

Although first identified as an RNA binding protein, the proliferation associated 2G4 protein, PA2G4, directly binds and stabilizes MYCN by protecting MYCN from proteasomal degradation (65). When PA2G4 is silenced in NB cells using siRNAs or a small molecule inhibitor WS6, MYCN protein levels are markedly reduced (65). WS6 treatment of NB cell lines completely blocked PA2G4-MYCN protein binding, and this competitive chemical inhibition results in a delay of tumorigenesis in the TH-*MYCN* NB mouse model (65).

Protein methylation is a post-translational modification recently identified to regulate protein stability. The protein arginine methyltransferase 5 (PRMT5) interacts with both MYC and MYCN proteins (66, 121). Silencing of PRMT5 in *MYCN*‐overexpressing NB cells or *MYC*-driven medulloblastoma cells leads to a decrease in MYCN and MYC protein levels and cell growth inhibition (66, 121). Tandem mass spectrometry analysis of immunoprecipitated MYCN protein in NB cells reveals several potential sites of arginine dimethylation on MYCN protein, suggesting that MYCN may be methylated by PRMT5 as a protection from proteasomal degradation (66). Treatment with the PRMT5 inhibitor EPZ015666 results in a decrease of MYC protein levels and medulloblastoma cell growth, which suggests that PRMT5 inhibitors are potential therapeutics for *MYC*- and *MYCN*-driven cancers.

### Targeting MYCN Cofactors/Coregulators

**Figure 4:**
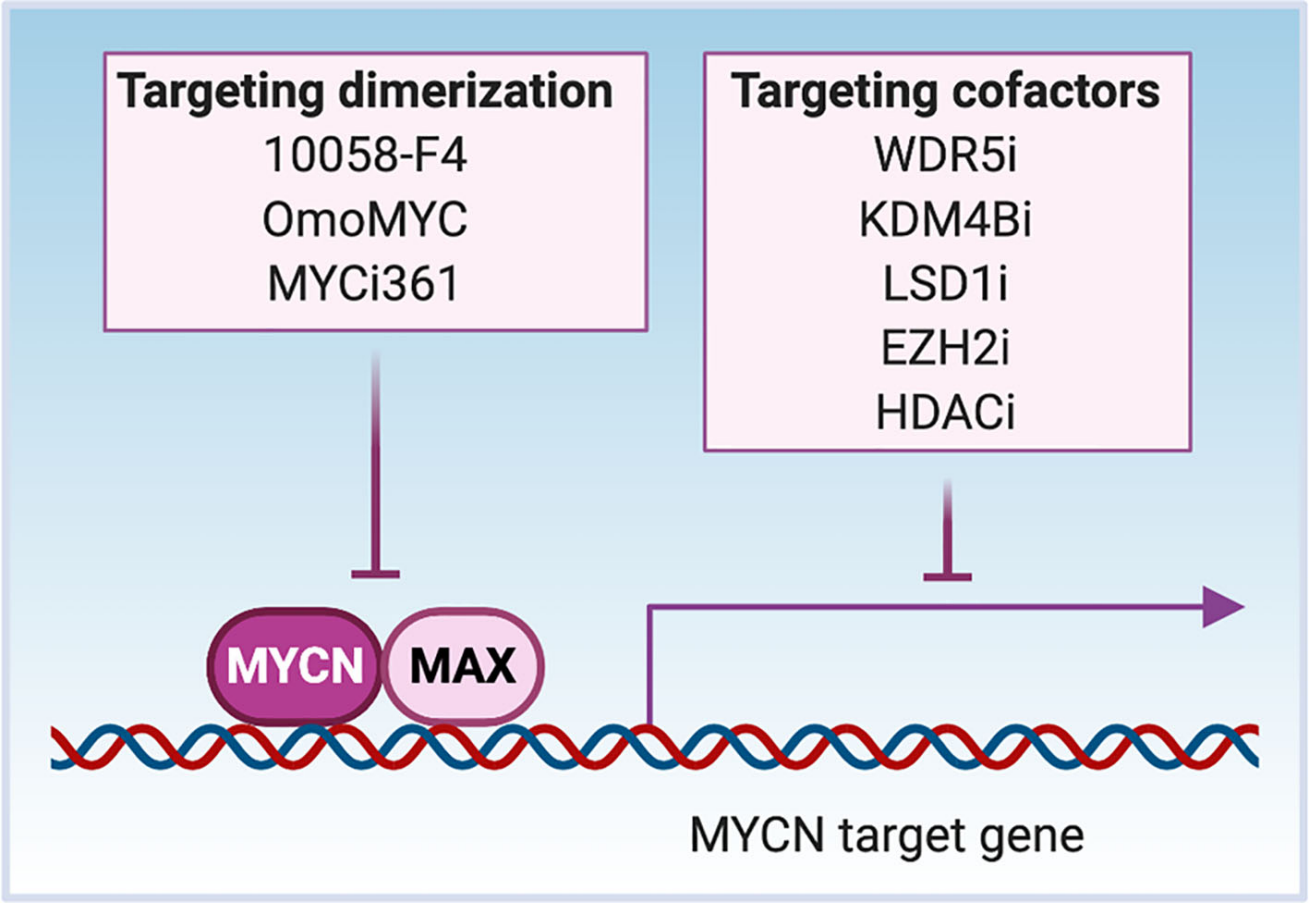
Targeting MYCN transcriptional activity. MYCN heterodimerizes with MAX to bind to the cis-genomic elements in DNA. MYCN interacts with cofactors WDR5 and KDM4B to activate gene transcription, while interacts with LSD1, EZH2 and HADCs to repress gene transcription through affecting chromatin status. Inhibitors such as 10058-F4, OmoMYC and MYCi361 disrupt the dimerization between MYCN and MAX to inhibit the DNA binding of MYCN. The treatment of cells with MYCN cofactor inhibitors (WDR5i, KDM4Bi, LSD1i, EZH2i or HDACi) inactivates MYCN transcriptional activity through regional epigenetic modification and/or opening or closing chromatin.

We have described critical regions that are needed for MYCN interactions with its cofactors/coregulators in the previous section and in **Figure 1**. As a TF, MYCN cooperates with other TFs to bind to DNA and recruit cofactors/coregulators to activate or repress gene transcription, making these protein partners potential targets to disrupt the transcriptional activity of MYCN (**Figure 4**). The enzymatic activity of many co-regulators makes them attractive drug targets.

The first identified mechanism through which MYCN functions as a TF is *via* heterodimerization with MAX. MYC-MAX complexes recognize E-box DNA sequences, and binding of the heterodimer to gene promoters activates transcription of downstream *MYCN*-related genes. Small-molecule inhibitors of MYC-MAX dimerization illustrate the importance of dimerization to MYC function (**Figure 4**). For instance, the peptidomimetic compound IIA4B20 exerts a strong inhibitory effect on MYC-MAX dimerization and DNA binding to functionally inhibit MYC-induced fibroblast transformation (122). The compound 10058-F4 binds to AA402-409 of MYC, which disrupts MYC-MAX dimerization of either c-MYC or MYCN. The treatment of *MYCN*-amplified NB cells with 10058-F4 leads to neural differentiation (123–125). Another known inhibitor of MYC-MAX dimerization is OmoMYC, a c-MYC derived mutant bHLH-LZ domain protein generated by substituting four amino acids within the c-MYC leucine zipper. When overexpressed, OmoMYC competes with MAX for binding to either c-MYC or MYCN and prevents MYC/MYCN proteins from binding to E-boxes and activating transcription (126, 127). The recently discovered MYC inhibitor 361 (MYCi361) binds to the HLH region of the MYC protein (AA366-378), disrupts MYC/MAX heterodimerization, enhances degradation of both MYC and MYCN, and suppresses MYC-dependent tumor cell growth *in vitro* and *in vivo* (128). The asymmetric polycyclic lactam, KI-MS2-008 stabilizes MAX homodimers, resulting in decreased MYC protein levels (129). Treatment of cancer cells with KI-MS2-008 suppresses *MYC*-dependent tumor growth *in vivo*. This is another example whereby altering the ability of MAX to dimerize with MYC functionally targets *MYC* (129). It will be interesting to evaluate whether either of these approaches affects MAX/MYCN interactions to inhibit the growth of *MYCN*-driven cancers.

MYCN has been shown to recruit several druggable cofactors with methylase and demethylase activity to regulate gene transcription, and cofactor inhibition provides a way to indirectly target MYCN (**Figure 4**). The histone H3K4 methyltransferase complex subunit WDR5 forms a protein complex with MYCN at the *MDM2* promoter that results in histone H3K4 trimethylation and activation of *MDM2* transcription (72). Treatment of NB cells with the WDR5 antagonist OICR9429 reduces MYCN/WDR5 complex formation and the expression of *MYCN* target genes, resulting in the inhibition of cell growth (72). When MYCN is overexpressed, it interacts with the H3K9me3/me2 demethylase KDM4B and recruits KDM4B to E-box containing regions to decrease H3K9me3 levels (73). Functional studies demonstrate that KDM4B acts as a MYCN co-activator to regulate MYCN signature genes. Knockdown of KDM4B decreases NB cell proliferation *in vitro* and NB xenograft growth *in vivo*, which provides proof-of-concept for the potential therapeutic efficacy of inhibiting KDM4B to target oncogenic MYCN signaling in cancers (73). MYCN has also been found to recruit co-repressors to suppress gene transcription. MYCN associates with EZH2, a methyltransferase and a member of the polycomb repressor complex 2 (PRC2) to repress the NB tumor suppressor gene CLU through a bivalent modification of the chromatin at the CLU promoter (78). The prevalence of this activity has not been evaluated. In *MYCN*-amplified tumors, MYCN increases levels of EZH2 and components of the PRC2 complex leading to increased activity of PRC2-mediated transcriptional repression primarily of differentiation associated genes. Genomic or pharmacologic inhibition of EZH2 suppresses NB growth *in vitro* and *in vivo* (130–132). MYCN also binds the lysine-specific histone demethylase 1A (KDM1A/LSD1) to repress gene transcription. LSD1 co-localizes with MYCN on promoter regions of CLU and CDKN1A, and the treatment with an LSD1 inhibitor restores the expression of these genes and suppresses NB cell growth (79). c-MYC interacts with histone methyltransferase EHMT2 to repress gene transcription, and knockdown of EHMT2 results in decreased tumor volume (133). EHMT2 is essential in NB cells and inhibition of EHMT2 using BIX-01294 decreased proliferation of NB cells and induced apoptosis (132, 134).

Acetylation and deacetylation of histones are key regulatory features of gene transcription and are potential targets that regulate MYCN transcriptional activity. MYCN recruits many HDACs (HDAC1, HDAC2 and HDAC5) to repress gene transcription (74, 135, 136). The histone acetyltransferase, GCN5, binds to MYC and MYCN proteins (67–69). *In vitro* luciferase assays show that MYC recruits GCN5 to activate gene transcription (70); however, few GCN5 specific inhibitors are available and have limited testing in NB cells (137).

### Targeting *MYCN* Downstream Targets

As a TF, MYCN regulates many target genes but the critical ones that mediate MYCN tumor initiating functions are not clear. One of the *MYCN* downstream targets that is under clinical evaluation is ornithine decarboxylase 1 (*ODC1*), the rate-limiting enzyme involved in polyamine synthesis (138, 139). In neuroblastoma, the expression levels of *MYCN* are strongly correlated with those of *ODC1*, and high levels of *ODC1* driven by *MYCN* amplification and overexpression are strongly associated with poor clinical outcome in NB patients (138). Treatment of TH-MYCN transgenic mice with the ODC inhibitor α-difluoromethylornithine (DFMO) prevents oncogenesis in hemizygous mice, while delaying tumor development in homozygous mice. Transient *Odc* ablation in hemizygous TH-*MYCN* mice permanently prevented tumor onset. This work indicates that *ODC* mediates an oncogenic function of *MYCN* that is important in tumor initiation and demonstrates the therapeutic potential of polyamine depletion strategies in NB (138, 139). A recent Phase II study of single agent DFMO as maintenance therapy in NB showed increased survival compared to historical controls for high-risk NB patients (140, 141).

The FACT (facilitates chromatin transcription) complex is another potential *MYCN* downstream target that is druggable. FACT facilitates transcriptional elongation on chromatin templates by binding and displacing the H2A/H2B dimer from nucleosomes, a process that is believed to be required for RNA polymerase II to pass through a nucleosomal barrier (142). MYC is confirmed to interact with a component of the FACT complex, the transcription elongation factor SSRP1 (4). SiRNA knockdown experiments demonstrate that expression of *FACT* and *MYCN* is controlled in a forward feedback loop, which drives *MYCN* transcription and protein stability (143). Inhibition of FACT using the small molecule curaxin compound CBL0137 results in a decrease of *MYCN* and *SSRP1* expression, as well as a markedly reduced NB tumor initiation and progression in the TH-MYCN mice especially when combined with standard chemotherapy (143).

### Targeting *MYCN* Synthetic Lethal Approach

Synthetic dosage lethality (SDL) is a genetic interaction in which the alteration of one gene, combined with the reduction in function of a second gene, results in lethality (144). SDL is an attractive therapy for cancer because inhibition of such a gene will only induce cell death in cells carrying the specific gene alteration. *MYCN* activates both proliferative and apoptotic cellular responses. Whether it promotes a net proliferative response is dependent on cooperating apoptotic factors such as the antiapoptotic protein BCL2 (145, 146). It has been demonstrated that *MYCN*-amplified neuroblastoma cells are highly sensitive to BCL2 inhibitors ABT-263 (navitoclax) and ABT-199 (venetoclax) (147). When screening for enhancers of ABT-199 sensitivity in *MYCN*-amplified NB, researchers found that the Aurora Kinase A inhibitor (alisertib) cooperates with ABT-199 to induce widespread apoptosis. This drug combination was more effective in killing *MYCN*-amplified NB cells *in vitro* and *in vivo* than either compound alone (147). Moreover, in *MYCN*-amplified NB, Polo-Like Kinase 1 (PLK1) and MYCN create a positive, feedforward activation loop essential for maintaining their high levels of expression (120). BCL2 antagonists have been shown to synergize with inhibitors of PLK1, such as BI6727 or BI2356 and may be an effective drug combination for NB over-expressing *MYCN* (120).

One of the mechanisms through which *MYCN* exerts its tumorigenic effect in NB is to activate transcription of genes involved in proliferation, including checkpoint kinase 1 (*CHK1*), an important regulator of the G1/S and G_2_/M checkpoints. This mechanism may contribute to the ability of *MYCN*-amplified NB tumors to become refractory to standard chemotherapy (148). Conversely, tumor cells lacking DNA damage checkpoints during tumorigenesis or during cytotoxic therapy are highly sensitive to additional genomic instability (149). *MYCN* induces replication stresses and DNA damage through excessive replication-fork firing. *MYCN*-overexpressing tumors are more sensitive to CHK1 inhibition (150, 151). Another cell cycle related synthetic lethality protein identified in *MYCN*-amplified NB is cyclin-dependent kinase 2 (CDK2) (152). Knockdown of CDK2 or treatment with the CDK2 inhibitor roscovitine induces apoptosis in *MYCN*-amplified neuroblastoma cell lines but not in those with *MYCN* single copy. Thus, inhibition of CDK2 is synthetically lethal to NB cells with overexpressed *MYCN* (152).

NB arising in adolescents and young adults is frequently associated with loss of function mutations in the alpha thalassemia X-linked (*ATRX*) gene (153, 154). Interestingly, *ATRX* mutations and *MYCN* amplification have never been observed in the same NB tumor, suggesting a potential synthetic lethal condition (153, 154). Doxycycline-induced overexpression of *MYCN* in *ATRX*-mutant NB cell lines showed a marked loss of tumor cells. Moreover, in the LSL-MYCN GEM of NB tumors failed to develop when LSL-MYCN : Dbh-iCre NB mice were crossed with *ATRX^flox^* mice demonstrating synthetic lethality between mutant *ATRX* and high levels of *MYCN* (154). This is an example of rare synthetic lethality between an inactivated tumor suppressor and an activated oncogene. *MYCN* has been shown to play an apoptotic role in cancer cells under certain circumstances (155). Thus, it is possible that under the stress of DNA replication, when *ATRX* is inactivated, high levels of *MYCN* induce an apoptotic cellular response. Therefore, ATRX targeting may be a therapeutic approach in *MYCN*-amplified NB tumors. Alternative strategies that increase MYCN protein levels may lead to an SDL situation in *ATRX*-mutant NB cells. Increasing MYCN levels may be achieved by interfering with critical components in the MYCN protein degradation pathway, such as HUWE1. HUWE1 ubiquitinates and directs MYCN degradation to the proteasome (61). Knockdown of the *HUWE1* gene impedes MYCN degradation and increases MYCN protein levels in NB cells (61). HUWE1 inhibitors such as BI8622 and BI8626 have been generated, but not tested in this situation.

Complementing experimental approaches to the identification of SDL in tumor cells, a recent computational approach utilized accumulating tumor genomic data to identify candidate SDL networks in various cancers (156). Synthetic lethality or ‘oncogene addiction’ offers an attractive therapeutic strategy for *MYCN*-driven cancers. Both bioinformatic analysis and high throughput drug screening can be used to identify novel-druggable synthetic lethal genes to which MYCN expressing cells are ‘addicted’.

### Prospect of Directly Targeting MYCN

Once considered undruggable, recent advances in chemistry and chemical genomics have begun to directly target transcription factors. Covalent reaction with their protein targets through cysteine residues is a known mechanism for many covalent drugs (157). A recent *in vitro* study that screened a library of cysteine-reactive covalent ligands, consisting of acrylamides and chloroacetamides, identified EN4. EN4 directly and covalently modifies the pure full-length c-MYC protein at cystine 171 (C171) of its intrinsically disordered region (158). In cells EN4 targets MYC interfering with MYC transcriptional activity. This reactive C171 on c-MYC is not conserved in MYCN. However, a similar screening approach could be used to identify small molecules that target cysteine residues in MYCN.

**Figure 5:**
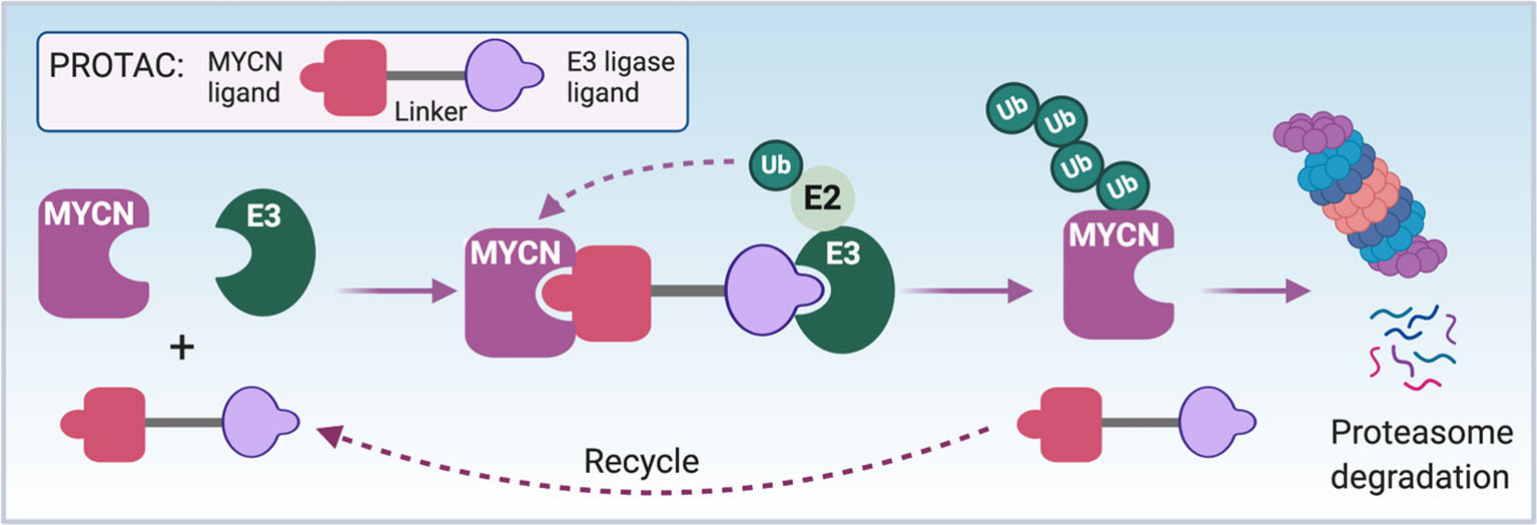
PROTAC strategy to directly target MYCN. The schematic illustrates the mode of action of a proteolysis targeting chimera (PROTAC) targeting MYCN. First of all, a bio-conjugatable analog of a MYCN binding ligand (such as modified 10058-F4 or MYCi361) will be conjugated to E3 ubiquitin ligase binding ligand through a linker to synthesize a MYCN PROTAC. The formation of MYCN-PROTAC-E3 ubiquitin ligase complex will result in a transfer of ubiquitin (Ub) to the lysine residues of MYCN by E2 ubiquitin-conjugating enzyme. Afterwards, the PROTAC will be released and reutilized, and the poly-ubiquitinated MYCN will undergo proteasome degradation. Notes: circled ‘Ub’ represents ubiquitin.

Proteolysis targeting chimeras (PROTACs) induced protein degradation is a recently developed therapeutic strategy, especially for undruggable targets (159). PROTACs are composed of three chemical elements: 1) a ligand binding to a target protein, 2) a ligand binding to E3 ubiquitin ligase, and 3) a linker for conjugating these two ligands (159). The small molecules 10058-F4, 7954-0035-G5, 10074-G5, JKY-2-169, MYCi361 and MYCi975 have been shown to bind to the HLH domain of MYC protein with some binding to MYCN protein as well (125, 128, 160). It may be possible to use these small molecules to develop PROTACs reagents to directly target and degrade the MYC/MYCN proteins (**Figure 5**). Intrinsically disordered region analysis of MYCN indicates that the MB II domain of MYCN is the most ordered region of MYCN having less flexibility and interacts with TRRAP (**Figure 1**). A small molecule or peptide screen to identify binders to the MBII region of MYCN would be another strategy to identify components needed to construct a MYCN PROTAC.

MiRNA are small RNA molecules that regulate their target gene expression at the post-transcriptional level and *via* their effects on the epigenetic machinery. Many miRNAs such as miR-34a, miR-375, miR-393-5p and let-7 are found to inhibit *MYCN* mRNA translation or target MYCN mRNA for degradation to suppress tumor cell growth (161–164). With more and more effective drug delivery systems for small interference RNA (siRNA) and miRNA being developed (165), directly targeting MYCN mRNA using miRNAs or siRNAs is a another approach for the treatment of *MYCN*-driven tumors. Indeed, the recent clinical findings which showed the systemic administration of a next generation antisense targeting the STAT3 TF decreased nuclear STAT3 levels in tumors are proof of principle that direct mRNA targeting of a transcription factor is feasible (166).

Targeting DNA amplification is another possible way to directly target *MYCN*. The genome-editing tool CRISPR-Cas9 is able to cut DNA at a targeted location and lead to cancer cell death if the targeted regions contain copy number gains. Whether this is clinically translatable is unknown. Pyrrole-imidazole (PI) polyamides when conjugated with DNA-alkylating agents could induce sequence-specific DNA alkylation to suppress target gene expression. A recent study showed that a *MYCN*-targeting PI- polyamide, MYCN-A3, binds to and alkylates DNA within the *MYCN* transcript, resulting in a decrease in *MYCN* copy number, downregulation of MYCN expression and suppression of NB growth *in vitro* and in xenografts (167). This indicates that the direct targeting of amplified *MYCN* at a genomic level is feasible. However, the feasibility of developing targeting approaches in pre-clinical models is only the first and sometimes the easiest step in the drug development pipeline.

## Conclusion

The oncogenic amplification and/or overexpression of *MYC* family genes occur in most human cancers, making MYC family oncogenes one of the most sought-after therapeutic targets. Here we specifically reviewed multiple pharmacological approaches to target *MYCN* by interfering with pathways that MYCN uses to drive oncogenesis. Indirect inhibitors of *MYCN*, such as the BET bromodomain inhibitor, the CDK7 inhibitor, the AURKA inhibitor, the HAUSP inhibitor and the ODC inhibitor have clearly shown benefit in suppressing *MYCN*-amplified tumor growth in the preclinical studies, and a few of these inhibitors including bromodomain inhibitor GSK525762, AURKA inhibitor MLN8237 and ODC inhibitor DFMO are being evaluated in the clinic for *MYCN*-driven cancers. Once again it is possible that combinatorial strategies that integrate these new approaches with standard chemo- and immunotherapy will lead to improved tumor control with less toxicity for patients.

## Author Contributions

ZL and CT conceptualized and wrote the manuscript while SSC, SC, and VV wrote sections of the manuscript. All authors contributed to the article and approved the submitted version.

## Conflict of Interest

The authors declare that the research was conducted in the absence of any commercial or financial relationships that could be construed as a potential conflict of interest.
